# Diversity and biochemical features of culturable fungi from the coastal waters of Southern China

**DOI:** 10.1186/s13568-014-0060-9

**Published:** 2014-08-30

**Authors:** Li Li, Purnima Singh, Ying Liu, Shenquan Pan, Guangyi Wang

**Affiliations:** 1 School of Environment and Energy, Peking University Shenzhen Graduate School, Shenzhen 518055, China; 2 Department of Biological Science, National University of Singapore, Singapore 117543, Singapore; 3 Tianjin University Center for Marine Environmental Ecology, School of Environmental Science and Engineering, Tianjin University, Tianjin 300072, China; 4 Department of Microbiology, University of Hawaii at Manoa, Honolulu 96822, HI, USA

**Keywords:** Marine-derived fungi, Diversity, Hydrolytic enzymes, Pelleterization

## Abstract

Fungi play a major role in various biogeochemical cycles of terrestrial and marine ecosystems. However, fungi in marine environments remain to be one of the most under-studied microbial groups. This study investigates the diversity of planktonic fungi from the coastal habitat off Pearl River Delta (China) using culture-dependent approach. A total of 22 fungi and 9 yeast isolates were recovered from 30 seawater and 2 sediment samples. Microscopic and ITS rRNA gene sequence analyses revealed that most of the fungi belonged to the phylum *Ascomycota* and *Basidiomycota* with a very small percentage (3%) of the subphylum *Mucoromycotina* of the Phylum *Zygomycota*. Most of these fungal isolates exhibited considerable production of extracellular enzymes, cellulase, lipase and laccase. Fungal isolates of two genera *Mucor* and *Aspergillus* sp. demonstrated pelletization capability over a wide range of pH, suggesting them as potential agents towards algae harvesting and wastewater treatment.

## Introduction

Coastal marine habitats have been characterized as the most variable, highly diverse and rich in primary production (Jickells [1998]; Danovaro and Pusceddu [2007]). The primary production in coastal habitats sometimes reaches very high levels resulting into availability of a great fraction of organic matter for consumers as detritus even after consumption of herbivores (Newell [1982]). Different types of microbes in coastal waters degrade a large proportion of this detritus actively (Manini et al. [2003]; Pusceddu et al. [2003]). Among these microbes, heterotrophic bacteria and archaea have been described for their degradation abilities towards such detritus to a greater extent (Moran and Miller [2007]; Mou et al. [2008]). In spite of being a significant component of coastal waters, the diversity and ecology of heterotrophic eukaryotes however has not been received much attention (Giovannoni and Stingl [2005]; Hallam et al. [2006]; Fenchel [2008]; Strom [2008]).

Among eukaryotes, fungi have been reported to exhibit as individual filaments or aggregates in coastal waters (Gutiérrez et al. [2010]). However, in comparison with terrestrial environments, fungi in the world’s oceans remain largely unknown (Gao et al. [2010]). Despite of a few reports on diversity of fungi from the oceans, the diversity and ecology of their planktonic forms (mycoplankton) have barely been explored (Richards et al. [2012]). Mycoplankton include free-living filamentous fungi, yeasts, fungal-like protists, and those associated with planktonic particles or phytoplankton (Wang and Johnson [2009]; Gao et al. [2010]).

Fungi are a key component of the biosphere, fulfilling a wide range of biogeochemical and ecological functions in natural environments (Christensen [1989]; Pang and Mitchell [2005]). They are best known as decomposers of organic matter and play major role in nutrient regeneration in the detrital ecosystems. The filamentous mycelia may greatly enhance the efficient mineralization of particulate organic matter (Tisdall and Oades [1982]; Damare and Raghukumar [2008]) and thus benefit the growth of planktonic microbial communities (Kiørboe and Jackson [2001]; Gutiérrez et al. [2010]). The biomass of planktonic fungi has been reported to be comparable with prokaryotes including both Bacteria and Archaea (Gutiérrez et al. [2011]).

Fungi occupy distinct ecological niches from that of bacterioplankton in detritus ecosystems with the ability to utilize large lignocellulose-predominated substrates with high C: N ratio (Newell [1994]; Raghukumar [2004]). They possess the ability to penetrate relatively persistent particulate detritus much more efficiently than bacterioplankton (Raghukumar [2004]). The endophytic fungi have been demonstrated to reside within marine plants intra or intercellularly, and produce a variety of bioactive and chemically active metabolites (Kaul et al. [2013]). The bioactive metabolites produced by endophytic fungi originate from different biosynthetic pathways and belong to different groups of terpenoids, steroids, quinones, phenols and coumarins (Kaul et al. [2013]). Therefore, the endophytes represent a potential chemical reservoir for anticancer, antioxidant, antiviral and insecticidal compounds for pharmaceutical and agrochemical industries. Two new benzopyranones, diaportheone A and B, were obtained via bioassay-guided isolation of the secondary metabolites from the endophytic fungus *Diaporthe* sp. P133 isolated from *Pandanus amaryllifolius* leaves (Bungihan et al. [2011]). These benzopyranones have been successfully used as antimicrobial compounds towards several microorganisms. One of the fungal isolate belonging to *Aureobasidium pullulans* has been exhibited as a reservoir of biotechnologically active products in previous reports (Chi et al. [2009]).

Considering the crucial role of planktonic fungi in versatile oceanic biogeochemical cycles, their diversity needs to be addressed from different ecosystems. The coastal habitats of Pearl River Delta, China have been highly productive ecosystems of China, being the major source for fishing industries. However, recently there has been an increased pollution level detected in these ecosystems which may further enhance detritus levels available for degradation. Therefore, the mycoplankton diversity studies from these still unexplored habitats may provide greater insight on potential fungal isolates playing significant role in ecological cycles of Pearl River Delta. This study is the first report on diversity of planktonic fungi based on culture-dependent approach from the coastal habitats of Pearl River Delta.. Potential fungal isolates were further investigated for the production of different extracellular enzymes such as laccase, lipase and cellulase in order to understand their active role in ecological cycles of coastal ecosystems.

## Materials and methods

### Sample collection and isolation of fungi

Seawater and sediment samples were collected from coastal marine habitats of Pearl River Delta region of China during March, 2012 (Table 1). These samples were carried in sterile, screw capped plastic bottles and bags immediately back to the laboratory for isolation. The isolation of fungi from the seawater samples was done within one hour of collection using the membrane filtration technique. Briefly, 15 ml triplicate water samples were filtered through sterile 0.45 μm cellulose ester membranes (Millipore, USA). These membranes were then placed on solid media plates, Malt Extract Agar (MEA), Sabouraud Dextrose Agar (SDA), Potato Dextrose Agar (PDA), Czapek Dox Agar (CDA) and Corn Meal Agar (CMA), supplemented with antibiotics (0.075% streptomycin and 0.05% ampicillin) to suppress bacterial growth. Sediment samples (0.1 g) were suspended in 10 ml sterile seawater and 100 μl of the resulting suspension was plated directly on the above media plates containing antibiotics. The plates were incubated at room temperature (28°C) and examined daily for the growth of fungi. Fungal colonies that developed were subcultured onto fresh MEA plate for pure, single colony isolation and identification. The identification of filamentous fungi was done by macroscopic and microscopic morphology (Additional file 1: Figure S1). Three promising strains (*Rhodosporidium* sp. PKU Y5, *Rhodotorula* sp. PKU Y7 and *Cladosporium* sp. PKU F16) have been deposited in China General Microbiological Culture Collection Center (CGMCC No. 2.5198, CGMCC No. 2.5199 and CGMCC No.3.17121).

**Table 1:** Details regarding colony forming units (CFUs) of fungal colonies on the media plates

**Sampling date**	**Location**	**Lat(°N)**	**Long (°E)**	**Habitat**	**Depth (m)**	**Temperature (°C)**	**Salinity (ppt)**	**Media**	**No. of colonies**	**CFU/L**
2012-03-05	Shenzhen Bay	22°31'19.776"	113°57'4.284"	Seawater	0	16.25	31.02	MEA	3	120
		22°31'19.776"	113°57'4.284"	Seawater	0	16.25	31.02	SDA	2	80
		22°31'19.776"	113°57'4.284"	Sediment	0	16.25	31.02	MEA	12	480
		22°31'19.776"	113°57'4.284"	Sediment	0	16.25	31.02	SDA	55	2200
	Daya Bay	22°35'34.224"	114°30'28.800"	Seawater	0	16.25	31.02	MEA	4	160
		22°35'34.224"	114°30'28.800"	Seawater	0	16.25	31.02	SDA	5	200
		22°35'34.224"	114°30'28.800"	Sediment	0	16.25	31.02	MEA	6	240
		22°35'34.224"	114°30'28.800"	Sediment	0	16.25	31.02	SDA	8	320
2012-03-21	Mirs Bay	22°29'44.02"	114°27'34.38"	Seawater	0	17.37	31.37	MEA	1	40
		22°29'44.02"	114°27'34.38"	Seawater	0	17.37	31.37	SDA	3	120
		22°29'44.02"	114°27'34.38"	Seawater	5	17.37	31.37	MEA	2	80
		22°29'44.02"	114°27'34.38"	Seawater	5	17.37	31.37	SDA	4	160
		22°29'44.02"	114°27'34.38"	Seawater	10	17.37	31.37	MEA	3	120
		22°29'44.02"	114°27'34.38"	Seawater	10	17.37	31.37	SDA	5	200
	Mirs Bay	22°31'33.14"	114°27'51.59"	Seawater	0	17.74	31.37	MEA	3	120
		22°31'33.14"	114°27'51.59"	Seawater	0	17.74	31.37	SDA	2	80
		22°31'33.14"	114°27'51.59"	Seawater	5	17.74	31.37	MEA	4	160
		22°31'33.14"	114°27'51.59"	Seawater	5	17.74	31.37	SDA	13	520
		22°31'33.14"	114°27'51.59"	Seawater	10	17.74	31.37	MEA	3	120
		22°31'33.14"	114°27'51.59"	Seawater	10	17.74	31.37	SDA	10	400
	Mirs Bay	22°31'32.02"	114°28'26"	Seawater	0	17.69	31.38	MEA	2	80
		22°31'32.02"	114°28'26"	Seawater	0	17.69	31.38	SDA	8	320
		22°31'32.02"	114°28'26"	Seawater	5	17.69	31.38	MEA	5	200
		22°31'32.02"	114°28'26"	Seawater	5	17.69	31.38	SDA	7	280
		22°31'32.02"	114°28'26"	Seawater	10	17.69	31.38	MEA	5	200
		22°31'32.02"	114°28'26"	Seawater	10	17.69	31.38	SDA	17	680
	Daya Bay	22°34'19.99"	114°31'30"	Seawater	0	19.24	31.00	MEA	2	80
		22°34'19.99"	114°31'30"	Seawater	0	19.24	31.00	SDA	3	120
		22°34'19.99"	114°31'30"	Seawater	5	19.24	31.00	MEA	2	80
		22°34'19.99"	114°31'30"	Seawater	5	19.24	31.00	SDA	7	280
		22°34'19.99"	114°31'30"	Seawater	10	19.24	31.00	MEA	3	120
		22°34'19.99"	114°31'30"	Seawater	10	19.24	31.00	SDA	12	480
	Daya Bay	22°34'34.37"	114°30'30.46"	Seawater	0	19.69	30.86	MEA	3	120
		22°34'34.37"	114°30'30.46"	Seawater	0	19.69	30.86	SDA	9	360
		22°34'34.37"	114°30'30.46"	Seawater	10	19.69	30.86	MEA	10	400
		22°34'34.37"	114°30'30.46"	Seawater	10	19.69	30.86	SDA	8	320

### Isolation and sequencing of ITS rRNA gene from the fungal isolates

All the isolated fungi were grown in MEB for 4–5 days for DNA isolation. Yeasts were grown in YPD (yeast extract peptone and dextrose) medium and shaken at 170 rpm for 3–4 days. Mycelia and cells were harvested, lyophilized and crushed in a mortar and pestle to fine powder. Isolation of DNA was carried out using the Ezup Soil DNA extraction kit (Sangon Biotech, China), following the manufacturer’s guideline. The small subunit ITS rRNA gene was amplified by polymerase chain reaction (PCR) in the DNA T100™ Thermal cycler (Bio-Rad, USA) using the ITS rRNA gene specific primers ITS1 (5’-TCCGTAGGTGAACCTGCGG-3’) and ITS4 (5’-TCCTCCGCTTATTGATATGC-3’) (White et al. [1990]). One microliter of DNA (~25 ng) was added to 50 μl reaction volume containing 25 μl of Taq PCR mix (Generay, China) , 23 μl dd water and 5 pmoles of each primer. The PCR program was run for initial denaturation step at 95°C for 3 min, followed by 35 cycles of 1 min at 94°C, 0.5 min at 50°C and 1 min at 72°C, and a final extension at 72°C for 5 min. The PCR products were purified using Gel DNA extraction kit (NewTopBio, China). Amplified products were transformed into *Escherichia coli* DH5α cells (Invitrogen, Carlsbad, CA, USA), following the manufacturer’s instructions. Transformants were grown overnight at 37°C in Luria-Bertani broth containing 100 mg of ampicillin. The presence of insert was confirmed by PCR with M13 forward and reverse primers. One ml of the broth containing the clone was added to 25 ml of PCR reaction mixture. PCR protocol included an initial hot start incubation (5 min at 94°C) followed by 34 cycles of denaturation at 94°C for 30 s, annealing at 55°C for 30 s, and extension at 72°C for 1 min followed by a final extension at 72°C for 5 min. Clones containing positive insert were further processed for plasmid isolation and purification using Millipore plasmid preparation kit (Millipore, USA). Clones containing positive insert were sent to BGI (Shenzhen, China) for sequencing analysis using M13 primers.

### Phylogenetic analyses

Forward and reverse sequences were edited and assembled using Chromas Pro version 1.34 (Technelysium Pty Ltd, Tewantia, Queensland, Australia). The final sequences were compared to the nucleotide sequences of reference organisms available in the GenBank database using Blastn (Altschul et al. [1990]). The ITS1-5.8S-ITS4 gene sequences obtained for the organisms were aligned with their closest match using the program, ClustalW (Thompson et al. [1994]). Gaps and ambiguously aligned sequences were removed manually from further analyses. Phylogenetic analyses were carried out using distance setting (Maximum parsimony) in MEGA 4 software (Tamura et al. [2007]) with 1,000 bootstrap replicates. The resulting ITS1-5.8S-ITS4 gene sequences were submitted to GenBank under the accession number of KC113282-KC113312.

### Qualitative assay for extracellular enzymes

The enzymatic activity of all the fungal isolates was analyzed in this study. The strains were screened initially using qualitative plate assay for three different enzymes (laccase, cellulase and lipase) by streaking them on the media plates, supplemented with specific substrates. Laccase activity was detected using MEA plates amended with ABTS (2, 2’-azino-bis-3-ethylbenzothiazoline-6-sulfonic acid) (Srinivasan et al. [1995]). The fungal isolates were grown on these media plates at room temperature for 4 days. Green color produced around the fungal colonies on media plates indicated laccase activity.

Cellulase (CMCase) activity was detected using carboxymethylcellulose (CMC)-MEA plates (Carder et al. [1986]). The CMC-MEA plates comprised 0.5% carboxymethylcellulose-sodium salt (CMC-Na), 1.0% glucose, 0.15% peptone, 0.01% yeast extract, 100% seawater, and 2.0% agar. After growing the fungal isolates for 3 days at room temperature, these plates were stained with 0.1% Congo red solution for 20 min at room temperature. The resulting plates were washed twice with 1.0 M NaCl, and were kept overnight at 4°C. Clear zones around the colonies indicated the CMCase activities (Nagano and Fraser [2011]; Carder et al. [1986]). Lipase activity was detected using MEA plates supplemented with 0.01% phenol red, 1% olive oil and 10 mM CaCl_2_. The pH was adjusted to 7.3-7.4 with 1.0 M NaOH (Singh et al. [2006]). After incubation for 3 days, a change in color from pink to yellow indicated the lipase activity.

### Quantitative analysis of enzymatic activities

On the basis of qualitative screening, four fungal strains (PKU F16, PKU F18, PKU Y5 and PKU Y8) were selected for quantitative studies. Fungal inoculums were prepared by growing isolates in MEB medium and yeast in YPD medium, respectively, for four days at 28°C. These inoculums were further subcultured to fresh MEB medium containing individual enzyme specific substrates and incubated on shaker at 30°C, 150 rpm for 4 days. All these experiments were performed in triplicates. Individual enzymes were quantified in the supernatants of the isolates.

The laccase activity was assayed using glycine-HCl (pH3.0) buffer and ABTS as substrate (Niku-Paavola et al. 1988). Five hundred μl of crude culture filtrate was incubated with equal volumes of buffer containing ABTS and laccase activity was measured at 405 nm. The enzyme units were expressed as μM of substrate transformed per minute per liter of culture filtrate i.e. as enzyme units per liter of culture filtrate (UL^−1^). In the absence of the enzyme activity, no increase in the rate of absorbance was observed.

Cellulase activity was assayed following the method described by Raghukumar et al. ([1994]). A reaction mixture containing 200 μl culture filtrate and 200 μl of 0.5% CMC in 0.05 M sodium phosphate buffer, pH 7 was incubated at 37°C for 30 min. To terminate the reaction, 1 ml of DNS (dinitrosalicylic acid) reagent was added to above reaction mixture and then was boiled for 5 min. CMCase activity was measured at 575 nm. One unit of CMCase activity was defined as the amount of enzyme liberating 1 μM of reducing sugar per minute under the above assay conditions.

For lipase assay, 6 mg *para*-nitrophenyl palmitate (pNPP), dissolved in 2 ml isopropanol and 18 ml 50 mM sodium phosphate buffer (pH 8) was used as the substrate. One ml each of culture filtrate and sodium phosphate buffer was added to 1 ml of the pNPP substrate solution. After incubating at 37°C for 30 min, the released product, pNP (*para*-nitrophenol) was measured at 410 nm. One unit of lipase activity was defined as the amount of enzyme liberating 1 μM of pNP per minute under these assay conditions.

### Fungal pelletization experiment

Three of the fungal isolates (*Mucor* sp., PKU F1), (*Aspergillus* sp., PKU F8) and (*Cladosporium* sp., PKU F14) were investigated for pellet formation in this study. The spore suspension from these species was obtained by rinsing the mycelia on media plate with distilled water containing 10% Tween 20. The number of spores in the suspension was counted using an optical microscope (Olympus BX53 Manual fluorescence microscope). The spore suspensions were added to the 250-mL Erlenmeyer flasks containing 50 ml of nutrient media (Malt Extract Broth). The pellet formation experiments were performed by placing these Erlenmeyer flasks on a horizontal shaker (100 rpm) at room temperature for 4 days. The pellet formation was analyzed at different spore concentrations and pH ranges (2–8).

## Results

### Culturable diversity

**Figure 1:**
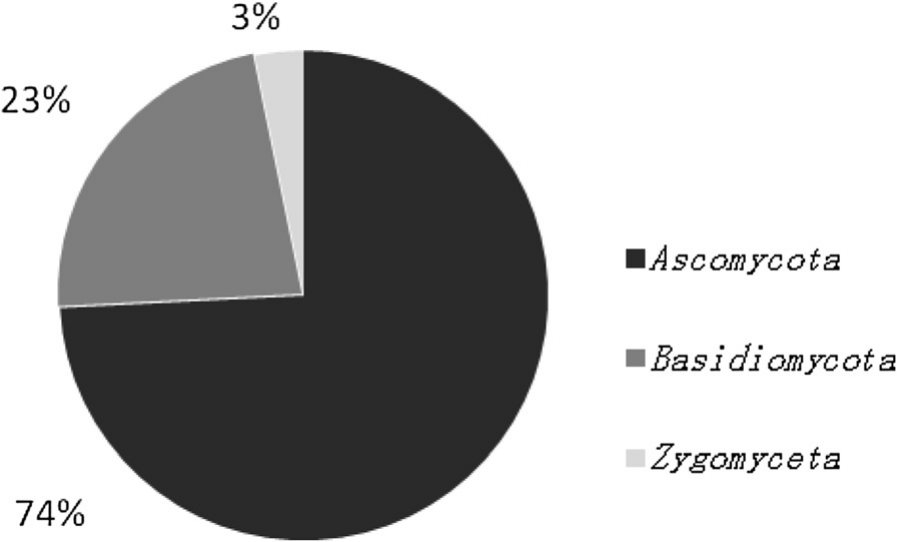
Phylum affiliation of culturable fungi with the existing sequences of NCBI database.

**Figure 2:**
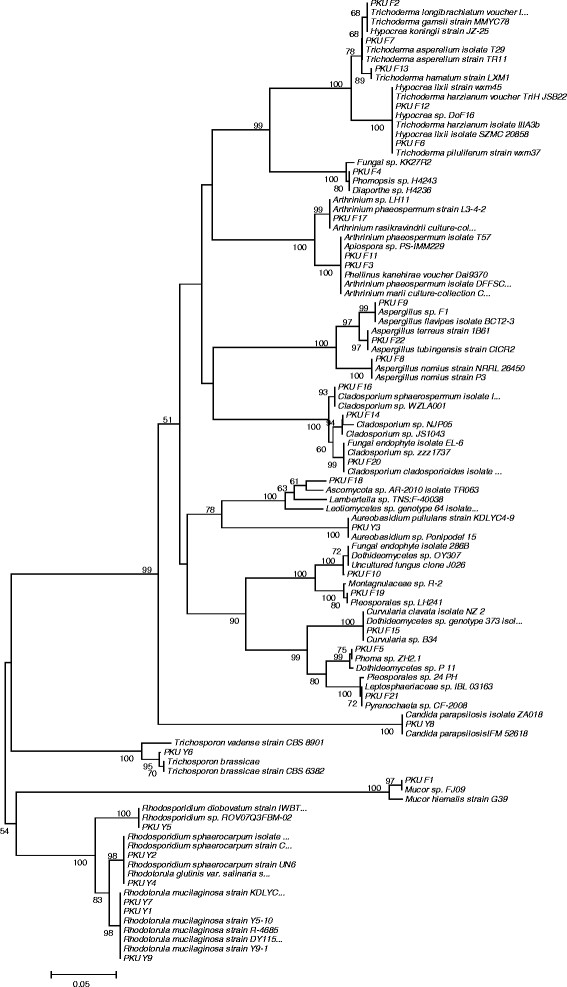
**NJ phylogenetic tree based on ITS rRNA genes from 22 fungi and 9 yeast derived from coastal ecosystems of Pearl River Delta.** Numbers at nodes indicate bootstrap values of neighborjoining analysis for 1,000 replicates (values below 50% not shown).

Fungi were isolated from all the seawater depths in the present study (Table 1). However, maximum numbers of fungal colonies were recovered from surface sediments (0 m depth) and seawater samples at 10 m depth. Fungi were also recovered from sediment samples using particle plating technique (Table 1). SDA was found to be better media than MEA for the isolation of fungi (Table 1). However, there was no statistical significance observed between different depths, media and the number of fungal colonies. A total of 22 fungi and 9 yeasts were isolated in this study. The fungal isolates mostly belonged to *Ascomycota*, *Basidiomycota* and *Zygomycota* based on ITS rRNA gene analysis (Table 2, Figure 1). Fungal isolates showing similarity with the phylum *Ascomycota* were dominating among above three, accounting for 74%. Members of *Basidiomycota* and *Zygomycota* made up for 23% and 3%, respectively (Figure 1). Of the Ascomycetes, isolates belonged to 13 genera i.e. *Aspergillus, Hypocraea, Arthrinium, Diaporthe, Phoma, Trichoderma, Dothideomycetes, Cladosporium, Curvularia, Pleosporales, Pyrenochaeta, Aureobasidium* and *Candida*. Isolates of Basidiomycota were affiliated with *Rhodotorula, Rhodosporidium* and *Trichosporon* sp. Only *Mucor* sp. of *Zygomycota* was identifed in this study (Table 2, Figure 2). All the fungal ITS rRNA gene sequences showed 100 or 99% identity with the existing sequences of NCBI database except PKU F18, showing 92% identity with *Ascomycota* sp. AR-2010 (Table 2).

**Table 2:** Phylogenetic affiliations of culturable fungi based on of ITS rDNA gene sequences

**Isolate ID**	**GenBank accession No.**	**Closest identified relative**		**Phylum of closest relative**	**Source of isolation of the closest relative**	**% Identity**
		**Species**	**(GenBank accession No.)**			
PKU F1	KC113282	*Mucor* sp. FJ09	(HQ019160)	*Zygomyceta*	Water hyacinth leaf	99
PKU F2	KC113283	*Hypocrea koningii* strain JZ-25	(HQ637343)	*Ascomycota*	Soil from Sichuan	99
PKU F3	KC113284	*Arthrinium phaeospermum* isolate T57	(FJ462766 )	*Ascomycota*	Not known	99
PKU F4	KC113285	*Diaporthe* sp. H4236	(GU595056 )	*Ascomycota*	Mangrove in China	98
PKU F5	KC113286	*Phoma* sp. ZH2.1	(FJ450059 )	*Ascomycota*	*Argyrosomus argentatus*	99
PKU F6	KC113287	*Trichoderma piluliferum* strain wxm37	(HM037939 )	*Ascomycota*	River water	99
PKU F7	KC113288	*Trichoderma asperellum* isolate T29	(JN108927)	*Ascomycota*	Rhizospheric soil	99
PKU F8	KC113289	*Aspergillus nomius* strain NRRL 26885	(JF824686)	*Ascomycota*	*Philanthus triangulum*	99
PKU F9	KC113290	*Aspergillus flavipes* isolate BCT2-3	(JQ082507 )	*Ascomycota*	Shark gills	100
PKU F10	KC113291	*Dothideomycetes sp. OY307*	(FJ571450 )	*Ascomycota*	Not known	99
PKU F11	KC113292	*Arthrinium phaeospermum* isolate DFFSCS004	(JX156350)	*Ascomycota*	Deep-sea sediments	99
PKU F12	KC113293	*Hypocrea lixii* isolate SZMC 20858	(JX173851)	*Ascomycota*	Hungarian vegetables	100
PKU F13	KC113294	*Trichoderma hamatum* strain LXM1	(GQ220703 )	*Ascomycota*	Saline-alkali soil	100
PKU F14	KC113295	*Cladosporium* sp. JS1043	(AM176680)	*Ascomycota*	Deep sea	100
PKU F15	KC113296	*Curvularia* sp. B34	(HQ696021)	*Ascomycota*	Moso Bamboo Seeds	99
PKU F16	KC113297	*Cladosporium sphaerospermum* isolate IJL07	( EU823317 )	*Ascomycota*	Soybean	99
PKU F17	KC113298	*Arthrinium* sp. LH11	(HQ832842 )	*Ascomycota*	Tea plants	99
PKU F18	KC113299	*Ascomycota* sp. AR-2010 isolate TR063	(HQ608095 )	*Ascomycota*	Soil	92
PKU F19	KC113300	*Pleosporales* sp. LH241	(HQ832825)	*Ascomycota*	Tea plants	100
PKU F20	KC113301	*Cladosporium cladosporioides* isolate 2728	(EU272532)	*Ascomycota*	Plant	99
PKU F21	KC113302	*Pyrenochaeta* sp. CF-2008	(EU885415)	*Ascomycota*	Corneal ulcer	99
PKU F22	KC113303	*Aspergillus terreus* strain 1B61	(KF572455)	*Ascomycota*	Geothermal soil	99
PKU Y1	KC113304	*Rhodotorula mucilaginosa* strain KDLYC24-1	(HQ909092 )	*Basidiomycota*	Not known	99
PKU Y2	KC113305	*Rhodotorula glutinis var. salinaria* strain ZH7-(7)	(FJ487944)	*Basidiomycota*	Mangrove in China	99
PKU Y3	KC113306	*Aureobasidium pullulans* strain KDLYC4-9	(HQ909088 )	*Ascomycota*	Not known	99
PKU Y4	KC113307	*Rhodosporidium sphaerocarpum* isolate 2223	(HQ670691)	*Basidiomycota*	Shrimp Culture	99
PKU Y5	KC113308	*Rhodosporidium diobovatum* strain IWBT-Y840	(JQ993385)	*Basidiomycota*	grapevines	99
PKU Y6	KC113309	*Trichosporon brassicae*	(NR_073251)	*Basidiomycota*	Not known	99
PKU Y7	KC113310	*Rhodotorula mucilaginosa* strain WC53-2	(EF190221)	*Basidiomycota*	Not known	99
PKU Y8	KC113311	*Candida parapsilosis* IFM52618	(AB109284)	*Ascomycota*	Braziland Japan	99
PKU Y9	KC113312	*Rhodotorula mucilaginosa* strain R-4685	(KF726105)	*Basidiomycota*	Aortic valve	100

### Enzymatic activities

**Figure 3:**
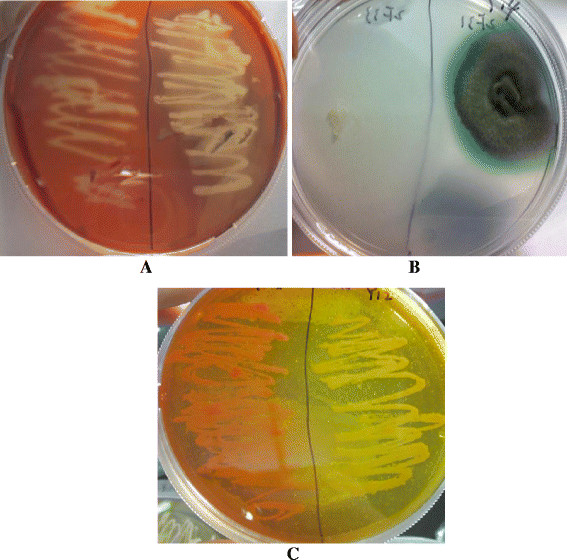
**Qualitative assay of enzyme activities of fungi on substrate specific media plates. A**. Plate assay for cellulose activities (PKU Y7 *Rhodotorula mucilaginosa* sp. (left) and PKU Y9 *Rhodotorula mucilaginosa* sp. showing good cellulose activity (rght)); **B**. Plate assay for laccase activities (PKU F11 *Arthrinium phaeospermum* sp. showing no laccase activity (left); PKU F16 *Cladosporium sphaerospermum* showing considerable laccase activities (right)); **C**. Plate assay for lipase activities on plate (PKU Y7 *Rhodotorula mucilaginosa* sp. showing little lipase activity (left); PKU Y9 *Rhodotorula mucilaginosa* sp. showing good lipase activity (right)).

Qualitative analyses of different enzymes demonstrated positive result for most of the fungal isolates (Table 3, Figure 3). The majority of fungal isolates (~84%) exhibited cellulase and lipase activities. In comparison, only a few of the isolates (~38%) showed positive laccase activity (Table 3, Figure 3). The quantitative analyses for production of these three enzymes were done for four of these fungal isolates (Table 4). *Cladosporium* sp. (PKU F16)*, Rhodosporidium* sp. (PKU Y5) and *Candida* sp. (PKU Y8) showed considerable production of lipase enzyme, reaching up to 21.94 U ml^−1^ for *Candida* sp. In contrast, cellulase production was not very high for any of these isolates, being maximum of 1.3 U ml^−1^ demonstrated by *Candida* sp. PKU F16 (*Cladosporium* sp.) and PKU F18 (*Ascomycota* sp.) were the best producers of laccase enzyme showing activities of 14.6 U ml^−1^ and 10.5 U ml^−1^ respectively (Table 4). All the isolates showed maximum production of these extracellular enzymes on 6^th^ day of their growth (Table 4).

**Table 3:** Qualitative assay of enzyme activities on media plates amended with specific substrates

**Isolate ID**	**Laccase (4**^**th**^**day)**	**Lipase (3**^**rd**^**day)**	**Cellulase (3**^**rd**^**day)**	**Source of isolation**	**Depth (m)**
PKU F1	-	+	+	Seawater	0
PKU F2	+	-	+	Seawater	0
PKU F3	+	++	++	Seawater	0
PKU F4	++	+	+	Seawater	0
PKU F5	-	++	-	Sediment	0
PKU F6	-	-	+	Seawater	0
PKU F7	++	+	++	Sediment	0
PKU F8	-	+	++	Sediment	0
PKU F9	-	++	++	Sediment	0
PKU F10	+	-	-	Sediment	0
PKU F11	+	++	+++	Seawater	10
PKU F12	-	++	++	Seawater	10
PKU F13	-	++	+++	Seawater	0
PKU F14	++	+++	++	Seawater	0
PKU F15	+	-	-	Seawater	10
PKU F16	+++	+++	++	Seawater	5
PKU F17	-	++	+	Seawater	10
PKU F18	+++	+	+++	Seawater	0
PKU F19	-	+	-	Seawater	0
PKU F20	++	++	++	Seawater	0
PKU F21	+	++	-	Seawater	0
PKU F22	-	+++	++	Seawater	0
PKU Y1	-	++	+	Seawater	0
PKU Y2	-	+	++	Seawater	0
PKU Y3	-	++	+	Seawater	10
PKU Y4	-	+	++	Seawater	0
PKU Y5	-	++	++	Seawater	10
PKU Y6	-	++	++	Seawater	5
PKU Y7	-	-	++	Seawater	0
PKU Y8	-	+++	+++	Seawater	0
PKU Y9	-	++	++	Seawater	10

**Table 4:** Quantitative assay of enzyme activities for four fungal isolates

**Isolate ID**	**Fungal genera**	**Growth** **date**	**Lipase (Umin**^**−1**^ **ml**^**−1**^**)**	**Cellulase (Umin**^**−1**^ **ml**^**−1**^**)**	**Laccase (Umin**^**−1**^ **ml**^**−1**^**)**
PKU F16	*Cladosporium sp.*	6th day	14.384 ± 0.994	0.843 ± 0.084	10.495 ± 1.300
		9th day	12.285 ± 0.772	0.443 ± 0.032	5.105 ± 0.943
PKU F18	*Ascomycota* sp.	6th day	6.605 ± 0.959	1.133 ± 0.096	14.609 ± 1.039
		9th day	4.630 ± 0.812	1.001 ± 0.049	7.581 ± 1.642
PKU Y5	*Rhodosporidium sp.*	6th day	17.174 ± 0.742	0.333 ± 0.040	-
		9th day	12.186 ± 1.705	0.371 ± 0.028	-
PKU Y8	*Candida sp.*	6th day	21.940 ± 1.582	1.311 ± 0.013	-
		9th day	18.767 ± 1.554	1.132 ± 0.029	-

### Fungal pellet formation

Among three fungal isolates i.e. *Aspergillus* sp. (PKU F8)*, Mucor* (PKU F1) and *Cladosporium* sp. (PKU F14), PKU F1 was found to be the best agent for pellet formation (Table 5, Additional file 1: Figure S2). The pH range of 6 and 8 were most suitable for all of these four isolates for pellet formation. Pellet sizes were comparative bigger with higher concentration of spore inoculums for *Mucor* sp. (Table 5). However, *Aspergillus* sp. did not show much variation with inoculum spore concentrations (Table 5). Maximum size of pellet of 7 mm was demonstrated by *Aspergillus* sp. (PKU F8) at pH 8.

**Table 5:** Details regarding pellet formation by the fungal isolates

**Isolate ID**	**Species**	**No. of spores (per L)**	**Pellet color**	**Different pH**			
				**PH = 2**	**PH = 4**	**PH = 6**	**PH = 8**
				**Pellet size(mm)**	**Pellet size(mm)**	**Pellet size(mm)**	**Pellet size(mm)**
PKU F1	*Muco*r sp. FJ09	4.00*10^6^	yellow	-	cotton- shaped	5	4
		1.60*10^7^	yellow	-	cotton- shaped	2 pellets (20*10)/(5*10)	1 pellet (20*10)
PKU F8	*Aspergillus nomius* strain	1.59*10^7^	white	<0.5	4-6	3	3-7
		6.34*10^7^	white	<0.5	1-2	3	4
PKU F14	*Cladosporium* sp.	3.85*10^8^	black	<0.1	0.5	0.5-1	1-2
		1.54*10^9^	black	-	-	-	1

## Discussion

Diversity of fungi has been reported from various environments such as freshwater (Gulis et al. [2006]), marine environments such as coastal waters (Gao et al. [2010]), deep-sea sediments (Damare et al. [2006]; Singh et al. [2010]), hypersaline waters (Buchalo et al. [2000]), methane hydrates (Lai et al. [2007]), oxygen deficient ecosystems (Cathrine and Raghukumar [2009]), mangroves and salt marshes (Hyde et al. [1998]; Raghukumar [2004]), and hydrothermal vents (Le Calvez et al. [2009]). This study is the first report on culturable diversity of fungi from coastal ecosystems of Pearl River Delta, China.

Despite of being isolated from coastal environment, the diversity of fungi was observed to be comparatively less, resulting into a total of 22 filamentous fungi and 9 yeast isolates. The isolated fungi belonged mostly to *Ascomycota*, *Basidiomycota* and at a very less percentage of *Zygomycota* (Table 2). Previous studies have also reported abundance of above fungal phylotypes in coastal seawater ecosystems (Gao et al. [2010]). The results obtained in the present study are also in concordance with earlier reports where surface water of coastal ecosystems has been reported to contain higher mycoplankton diversity compared with open-ocean ecosystems (Gao et al. [2010]). However, use of only culture-dependent approach in this study for estimation of diversity may have limited the isolation of other genera of fungi which are present as uncultivable forms in the oceanic habitats. Previous studies have reported a greater percentage of fungal diversity when assayed using combined approach of culture-dependent and culture-independent methods (Singh et al. [2012]). Therefore, a detailed study on fungal diversity from Pearl River Delta using both the approaches may provide a greater insight on hidden, still unexplored mycoplankton communities in future. The diversity of fungi has also been reported to vary with the nutrient contents of any particular habitat, which can be available as detritus for the fungal population (Newell [1982]).

Isolation of fungi was attempted using five different media (MEA, SDA, CMA, PDA and CMA). However, fungal isolates were recovered only on two media i.e., MEA and SDA (Table 3). In contrast, the fungal isolates were obtained with all the above five media during isolation from deep-sea sediments by Singh et al. ([2012]). Most of the isolates affiliated with the existing sequences of NCBI data base at the percentage identity of 100 or 99. However, the 92% identity of the fungal sequence PKU F18 with *Ascomycota* sp., suggests its probability of being novel. On the contrary, the insufficient database for ITS sequences may also be one of the reasons for such low similarity values (Zachow et al. [2009]; Anderson et al. [2003]).

The production of extracellular enzymes i.e., laccase. cellulase and lipase was demonstrated by most of the fungal isolates in the present study, suggesting their active role in various ecological cycles of coastal ecosystems off China. Laccase in one of the important lignin degrading enzymes, demonstrated to decolorize a range of dyes and toxic industrial effluent in earlier reports (Nyanhongo et al. [2002]). The fungal isolates PKU F16 and PKU F18, showing considerable quantitative production of laccase in the present study render them possible candidates for industrial application (Table 4). Cellulose is the most common substrate present in the seawater column in the form of plant biomass. It is found in nature exclusively in plant cell walls, although it is produced by some animals also e.g., tunicates and few bacteria (Lynd et al. [2002]). Fungi are well known agents of decomposition of organic matter composed of cellulosic substrate in particular (Lynd et al. [2002]). Therefore, cellulase production by the fungal isolates in the present study suggest their active involvement in the mineralization and leaf litter degradation in the coastal sea water habitats. Lipases are ubiquitous enzymes of considerable industrial and physiological significance. They catalyze the hydrolysis of triacylglycerols to glycerol and free fatty acids. Lipases are widely used in the processing of fats and oils, detergents and degreasing formulations, food processing, the synthesis of fine chemicals and pharmaceuticals, paper manufacture, and production of cosmetics, and pharmaceuticals (Rubin and Dennis [1997a], [b]; Kazlauskas and Bornscheuer [1998]). Lipase production up to 21.9 U ml^−1^ by *Candida* sp. in the present study reveals the potential of this fungus towards lipolytic degradation abilities. Additionally, the optimization of media and nutrient condition for enhanced lipase production from above isolate may be applied in future to maximize their industrial application and commercialization.

Fungi have been characterized widely for their efficient role in pellet formation towards harvesting of algae and wastewater treatment (Zhou et al. [2012]). The microalgae cells can be processed into a broad spectrum of biofuels by the transesterification process. These biofuels include biodiesel, green diesel and gasoline, being produced by transformation of algal biomass using various technologies (Chisti [2007]). However, many challenges have restricted the development of algal biofuel technology to commercial practicality that could allow for sustainable production and utilization (Brennan and Owende [2010]). Among these, one is the harvesting process of algae, which can be improved by application of agents, causing aggregation of algal cells. The pellet formation capabilities exhibited by three of the fungal isolates in the present study (Table 5, Additional file 1: Figure S2) opens new avenues for their efficient utilization towards algae harvesting and wastewater treatment. In addition, the pellet formation capabilities of fungi have been shown to be affected widely by various factors such as pH, inoculum concentration and trace metals (Zhou et al. [2000]). A co-cultivation method using fungi of the present study with algal strains under different pH, trace metals and inoculum concentration for efficient optimization of the harvesting process is suggested for future studies.

In conclusion, the fungal diversity obtained from this study was low. The fungal isolates belonged to three major phyla i.e., *Ascomycota*, *Basidiomycota* and *Zygomycota*, with *Ascomycota* being the dominant forms. The different qualitative as well as quantitative levels of extracellular enzymes produced by these isolates suggests them as significant component of the ecological cycles of coastal ecosystems off Pearl River Delta. Finally, the production of enzymes and pellet formation abilities of a few fungal isolates indicate their possible utilization in biotechnological industries.

## Competing interests

The authors declare that they have no competing interests.

## Author’s contributions

GW & PS conceived and designed the experiments; LL & YL performed the experiments; PS & LL analyzed the data; GW & PS contributed reagents/materials/analysis tools; PS, LL, GW & SQP wrote the paper. All authors read and approved the final manuscript.

## Additional file

## Supplementary Material

Additional file 1: Figure S1. Microscopic photograph of fungal isolates. **Figure S2.** Pellet formation by different fungal isolates using spores as inoculums at PH 6. Click here for file
