# Supplementary material for: Diversity and biochemical features of culturable fungi from the coastal waters of Southern China
Source: AMB Express. 2014 Aug 30;4:60. doi: 10.1186/s13568-014-0060-9 (PMC4230900; doi:10.1186/s13568-014-0060-9)
Supplement: Additional file 1: Figure S1. — Microscopic photograph of fungal isolates. Figure S2. Pellet formation by different fungal isolates using spores as inoculums at PH 6. [file s13568-014-0060-9-S1.pdf]

Supplementary Information for

Diversity and Biochemical Features of Culturable Fungi from the Coastal Waters of Southern China

Li Li[1,2], Purnima Singh[1], Ying Liu[1], Shen-Quan Pan[2] and Guangyi Wang[1,3,4]*

*To whom correspondence should be addressed.

Email: gywang@tju.edu.cn/guangyi@hawaii.edu

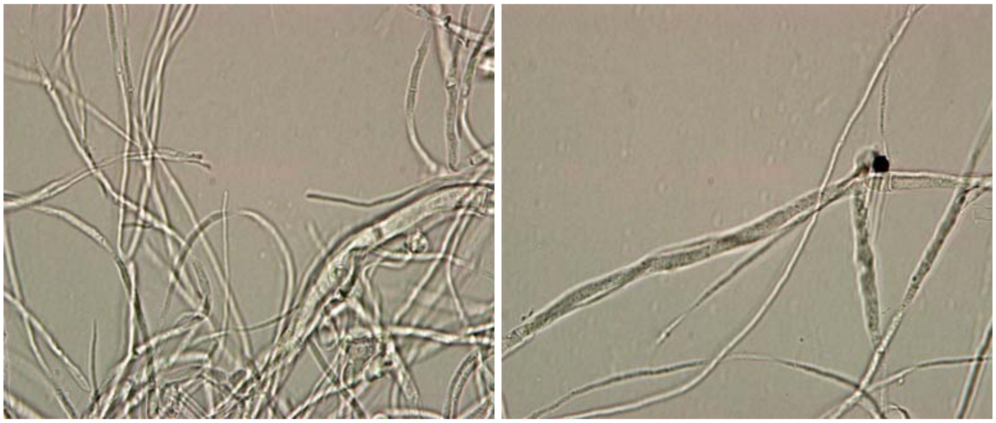

a. PKU F3, *Arthrinium phaeospermum* sp.

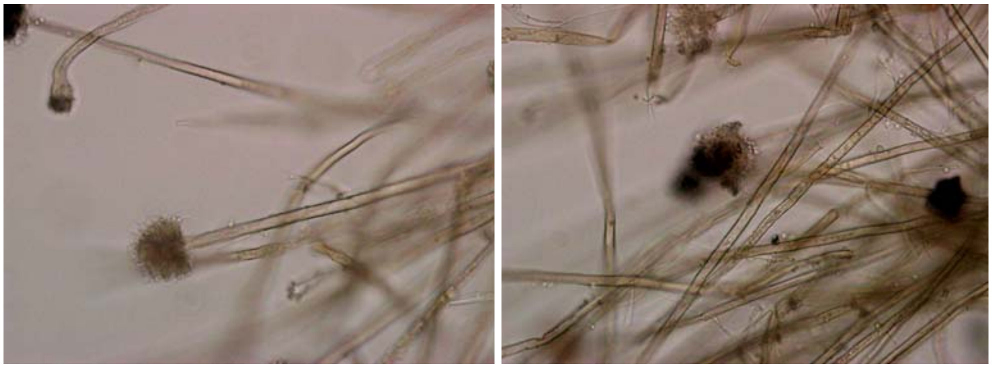

b. PKU F9, *Aspergillus flavipes* isolate sp.

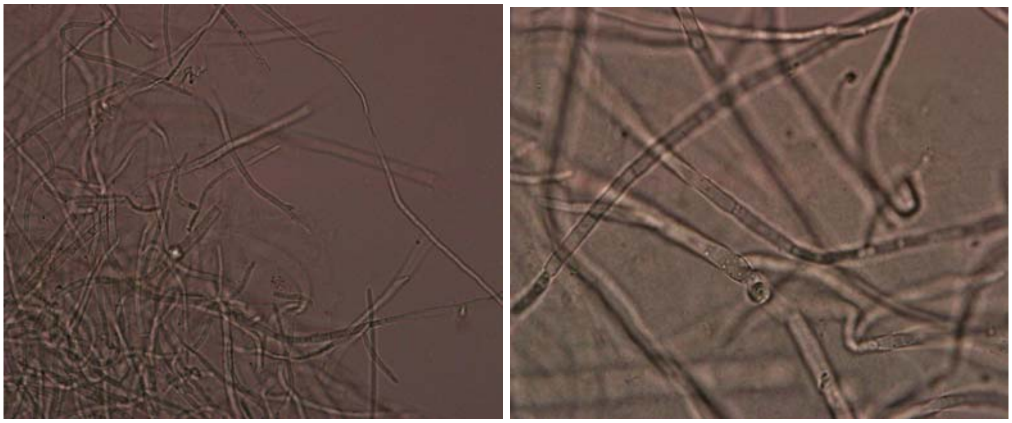

c. PKU F10, *Dothideomycetes sp*.

Supplemental Figure S1. Microscopic photograph of fungal isolates

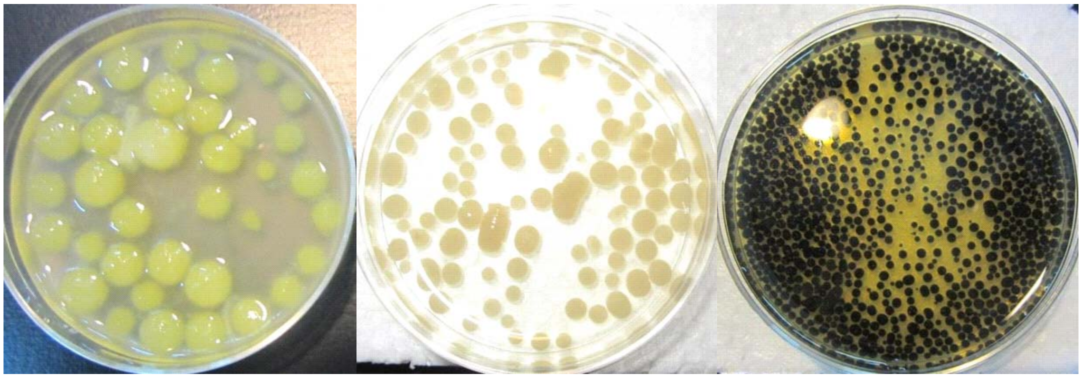

a) PKU F1 **b) PKU F8** **c) PKU F14**

Supplemental Figure S2. Pellet formation by different fungal isolates using spores as inoculums at PH 6
